# Empirical testing of hypotheses about the evolution of genomic imprinting in mammals

**DOI:** 10.3389/fnana.2013.00006

**Published:** 2013-04-30

**Authors:** David G. Ashbrook, Reinmar Hager

**Affiliations:** Computational and Evolutionary Biology, Faculty of Life Sciences, University of ManchesterManchester, UK

**Keywords:** genomic imprinting, parent-of-origin effects, evolution, coadaptation, recombinant inbred strains

## Abstract

The close interaction between mother and offspring in mammals is thought to contribute to the evolution of genomic imprinting or parent-of-origin dependent gene expression. Empirical tests of theories about the evolution of imprinting have been scant for several reasons. Models make different assumptions about the traits affected by imprinted genes and the scenarios in which imprinting is predicted to have been selected for. Thus, competing hypotheses cannot readily be tested against each other. Further, it is far from clear how predictions about expression patterns of genes with specific phenotypic effects can be tested given current methodology of assaying gene expression levels, be it in the brain or in other tissues. We first set out a scenario for testing competing hypotheses and delineate the different assumptions and predictions of models. We then outline how predictions may be tested using mouse models such as intercrosses or recombinant inbred (RI) systems that can be phenotyped for traits relevant to imprinting theories. Further, we briefly discuss different molecular approaches that may be used in conjunction with experiments to ascertain expression patterns of imprinted genes and thus the testing of predictions.

## Introduction

Genomic imprinting refers to differential gene expression characterized by either complete or partial silencing of either the paternally or maternally inherited allele (Barlow, 2011; Abramowitz and Bartolomei, 2012). There are several mechanisms by which imprinting can occur, chiefly DNA methylation (Strogantsev and Ferguson-Smith, 2012) and post-translational histone modifications, but other chromatin-, transcription- and ncRNA-mediated mechanisms have also been implicated (Kacem and Feil, 2009). However, imprinting effects caused by different mechanisms may manifest in the same way phenotypically, thus they are likely to be subject to the same selective pressures and therefore may be explained from an evolutionary perspective together (Wolf et al., 2008b). Since imprinting was discovered almost 30 years ago (McGrath and Solter, 1984; Surani et al., 1984), a number of hypotheses have been developed in an attempt to explain the evolution of uniparental or differential gene expression. In this quest one should differentiate between hypotheses that seek to explain the origins of imprinting, such as the host-defense hypothesis (Barlow, 1993; Walter and Paulsen, 2003; Haig, 2012), and those that seek to explain the selective pressures that fix and maintain genomic imprinting in a population. It is this second set of hypotheses that will be the subject of this perspective.

## Hypotheses for the evolution of genomic imprinting: their assumptions and predictions

Given that genomic imprinting renders gene expression in essence hemizygous and thus removes diploidy at a locus, any evolutionary theory needs to identify clear selective advantages to balance the cost of exposing the organism to potentially detrimental recessive alleles (Otto and Goldstein, 1992; Wilkins and Úbeda, 2011). Thus, we need to be able to explain how this fitness cost is outweighed by a fitness gain from imprinting, why particular genes are imprinted and why the vast majority of genes are not (Wilkins and Haig, 2003). In addition, any theory needs to account for the temporal and spatial patterns of imprinting, for example the strong effect on placental and brain phenotypes (Li et al., 1999). Here, we will examine three of the most widely supported hypotheses, namely the kinship, coadaptation and sexual conflict hypothesis, respectively.

## The kinship hypothesis

The kinship hypothesis, originally formulated as the conflict hypothesis, is the most widely accepted and empirically supported hypothesis (Moore and Haig, 1991; Haig, 1997; Wilkins and Haig, 2003; Trivers and Burt, 2006). In its original form the conflict hypothesis suggested that imprinting evolved due to the asymmetrical fitness consequences of maternal investment for maternally versus paternally derived alleles caused by relatedness asymmetries between paternal and maternal alleles in offspring (Haig and Westoby, 1989; Moore and Haig, 1991; Haig, 1997) The conflict hypothesis has been expanded into a wider kinship hypothesis, where the influence of all maternally related kin is weighed against the influence of all paternally related kin (Wilkins and Haig, 2003; Úbeda and Gardner, 2012).

The kinship hypothesis makes several assumptions (Table 1), two of which are more readily testable. Firstly, relatedness asymmetries arise when females breed with multiple males such that offspring have the same mother but different fathers. If females mated with a single male all offspring of consecutive broods would be equally related, and therefore there would be no conflict. Vrana and colleagues (1998) investigated predictions of the kinship hypothesis in crosses between largely monogamous *Peromyscus polionotus* and polygynous *P. maniculatus* and found parent-of-origin dependent growth differences in support of the kinship hypothesis. However, in contrast to predictions, imprinting at select loci was maintained in *P. polionotus*, which may either be explained by a lack of selective pressure to remove ancestral imprinting or the species may not be truly monogamous (Wilkins and Haig, 2003). Secondly, the kinship hypothesis assumes that there is a differential cost of expression of the gene in offspring for the parents, such that the costs fall more heavily on one than the other. Generally, the costs of parental investment to females are greater than those to males. This may be testable since different levels of imprinting, i.e., the degree to which differential expression exists at loci influencing parental investment, would be expected in species where the mother is the primary carer compared with species where the parents share offspring care. One testable prediction is that maternal expression is favored if a gene has a positive fitness effect when maternally derived but a negative effect when paternally derived, and vice versa for paternal expression. For example, increased maternal provisioning will have a positive fitness effect on the offspring, but may have a negative fitness effect on the mother's residual reproductive success. Since the current offspring are clearly related to their father, but the mother's future offspring are unlikely to be, genes that increase maternal provisioning are predicted to be paternally expressed. Similarly, since all offspring are by definition related to their mother, maternal provisioning will be decreased by maternally expressed genes to maintain the residual reproductive success of the maternal genotype. The predicted phenotypic effects of paternally and maternally expressed genes all assume that these genes, when expressed in offspring, can influence the level of maternal investment, e.g., through solicitation behavior. Another testable hypothesis that has been put forward for the wider kinship hypothesis is that biallelic expression may replace imprinting in aging adults, due to a reduction of conflict in older individuals (Úbeda and Gardner, 2012).

**Table 1:** **Key hypotheses for the evolution of genomic imprinting, with their assumptions and testable predictions**.

	**Kinship**	**Coadaptation**	**Intralocus sexual conflict**
Assumptions	Relatedness asymmetries arise from females that breed with multiple males.	Mother is the primary care giver.	Selection will favor different alleles in males and females.
	There is a differential cost of expression of the gene between maternally and paternally related individuals, such that the costs falls more heavily on one than the other.	Offspring genotype affects interactions with their mother.	Imprinting occurs during gametogenesis in the parent, and therefore is a result of sexual dimorphism.
		Genes controlling maternal phenotype may affect offspring phenotype either by pleiotropy or by linkage disequilibrium.	Sexually dimorphic imprinting from the parents can also have sexually dimorphic reading in the offspring.
Predictions	Maternal expression is favored if a gene would have a positive fitness effect when maternally derived but a negative effect when paternally derived, and vice versa for paternal expression.	The incidence of imprinting should be higher in taxa in which mother-offspring interactions have a greater effect on offspring fitness.	Paternal alleles are only expressed in males and maternal alleles are only expressed in females or imprinting will be in the direction of the strongest selection.
	The effects of paternally and maternally expressed genes expressed in offspring can influence the level of maternal investment.	The maternal genome should have greater control over imprinting.	
	Biallelic expression shown during development may replace imprinting in adults.	More genes will be maternally than paternally expressed.	Sexually selected traits should show imprinting.
Summary	Genomic imprinting is a result of conflicting benefits to maternally related kin and paternally related kin.	Genomic imprinting increases offspring fitness by increasing integration of coadapted maternal and offspring traits.	Genomic imprinting evolved due to different alleles being selected for in males or females at a given locus.

## Coadaptation hypothesis

The coadaptation hypothesis, similarly to the kinship hypothesis, concentrates on reproduction and development but suggests that coadaptation between offspring and mother, and not conflict, is responsible for imprinting, in particular the prevalence of maternally expressed genes (Wolf and Hager, 2006). In this scenario, genomic imprinting increases offspring fitness by increasing the integration of coadapted maternal and offspring traits and will therefore be favored by selection. The assumptions of the model are firstly that the mother is the primary care giver (although the model can equally well be applied to scenarios where the father is the primary care giver). Secondly, the model assumes that both offspring and maternal genotype affect offspring fitness through influencing traits involved in mother—offspring interactions. Genes controlling maternal phenotype may affect offspring phenotype either by pleiotropy (the same gene affects both offspring and maternal phenotype) or by linkage disequilibrium between the gene affecting maternal phenotype and the gene affecting offspring phenotype, such that they are inherited together. Since imprinting has predominantly been reported in mammals (Renfree et al., 2013) this assumption is well founded.

The coadaptation hypothesis predicts that more genes will be maternally than paternally expressed, as is the case for placentally expressed genes (Wagschal and Feil, 2006), but more recent publications have suggested that there is a more balanced level of imprinting (Wang et al., 2011). A second prediction is that the incidence of imprinting should be higher in taxa in which mother-offspring interactions have a greater effect on offspring fitness. The coadaptation hypothesis is supported by evidence that mouse pups are better provisioned by foster mothers of the same strain as their natural mothers, suggesting a coadaptation between offspring and maternal phenotype (Hager and Johnstone, 2003, 2005). While in humans the number of paternally expressed genes is greater than maternally expressed genes (81 vs. 95), this pattern is reversed in mice (64 vs. 47; geneimprint.com March 2013). This inconsistency may be taken as evidence against the coadaptation hypothesis, however, this theory focuses on traits involved in mother/offspring interaction and thus a subset of all imprinted genes.

## Intralocus sexual conflict hypothesis

The intralocus sexual conflict hypothesis suggests that genomic imprinting evolved due to different alleles being selected for in males and females at a given locus. Since all fathers and mothers are, by definition, reproductively successful, high-fitness paternal traits will be passed on to sons and high fitness maternal traits are transmitted to daughters. Thus, selection will favor so-called modifier loci that silence maternally inherited alleles in males and vice versa. In other words, imprinting is predicted to evolve because it mitigates intralocus sexual conflict. A further assumption is that while the imprinted gene is expressed in the offspring, the actual process of imprinting occurs during gametogenesis in the parent, and therefore is a result of sexual dimorphism (Day and Bonduriansky, 2004). It is predicted that, in some cases, this would lead to sexually dimorphic imprinted genes, where paternal alleles are only expressed in males and maternal alleles are only expressed in females. For example, if there is a reproductive advantage for males to be larger, but for females to be smaller, then the genes influencing size should be paternally expressed in males but maternally expressed in females. It may be difficult to distinguish this form of sexually dimorphic expression from others, such as sex chromosome dependent expression. If sexually dimorphic imprinting is not possible then the expression would be expected to be in the direction of the greater reproductive advantage (Day and Bonduriansky, 2004). On the other hand, if size has no influence on the reproductive success of females but still improved male fitness, then the genes would be expected to be paternally expressed. Further, this hypothesis predicts that many sexually selected traits should show imprinting. Since both growth and behavior can be sexually selected this fits well with the currently found over-representation of imprinted genes in the placenta and brain, and investigating other sexually selected phenotypes, such as mating behavior, may provide evidence for this hypothesis. With regard to empirical support, while evidence of sex dependent imprinting has been found, the results do not confirm the predicted patterns (Hager et al., 2008a), possibly due to the selective regime applied to the study model.

## Hypothesis testing

While the above three hypotheses represent currently favored views on the evolution of imprinting, several others were developed in the 1990's, e.g., the evolvability model (Beaudet and Jiang, 2002), evolution of X-chromosomal imprinting (Iwasa and Pomiankowski, 1999, 2001), and are discussed elsewhere (e.g., Ohlsson et al., 1995). Each of the above hypotheses makes explicit predictions with regard to the expression pattern (paternal or maternal expression) of genes that have specific phenotypic effects: the conflict hypothesis predicts expression patterns of genes that have asymmetrical fitness consequences for maternally vs. paternally derived genes, the coadaptation hypothesis predicts that at loci where coadaptation has important fitness consequences, maternal expression is predicted if the mother is the primary care giver. Finally, the intralocus sexual conflict hypothesis predicts that imprinting evolves at loci under sex-specific selection.

Testing these predictions empirically is challenging for a number of reasons. First, one would have to unequivocally identify loci that meet the assumptions e.g., being under sex-specific selection. To achieve this, the phenotypic effects of the loci would have to be established and then a call would have to be made at what stage in development (e.g., early or adulthood) and what tissue the pattern of expression will be measured and by which method.

What constitutes support for a given hypothesis and when would it be refuted? Taking the conflict hypothesis as an example, several imprinted loci that affect growth follow the predicted pattern of growth enhancers showing paternal expression and inhibitors maternal expression (see Table 4.2 in Trivers and Burt, 2006). Nonetheless, a significant number of loci do not support the predictions made. Thus, one may conclude that one way by which imprinting has evolved at loci conforming to the predicted patterns is due to conflict as postulated by the kinship hypothesis (say hypothesis A). Because alternative hypotheses (B and C) and their predictions make different assumptions about the phenotypic consequences of imprinted loci we cannot directly test hypotheses A against B and C, which, however, would be the hallmark of good science (Nakagawa and Cuthill, 2007).

In the absence of several hypotheses based on the same assumptions and making competing predictions the best we can do is test each hypothesis individually following the steps outlined above. It will be challenging to convincingly establish whether a given locus may be under sex-specific selection, has asymmetrical fitness consequences or plays a key role in coadaptation. Further, all of such tests would focus only on a select number of loci and is thus limited in the generality of its conclusions. A genome-wide approach may thus be more appropriate in the first instance covering all potential loci and their expression patterns (e.g., Hager et al., 2008a; Wolf et al., 2008a). Any of the above hypotheses may be true for specific loci whose expression was measured at given stage in development in a specific tissue. The crux is that the same loci may show a different imprinting pattern at other time points or in different tissues. This variability has been demonstrated in a number of genome-wide analyses (Cheverud et al., 2008; Wolf et al., 2008a). Whether or not these results reflect the possibility that imprinting may have evolved for different reasons at different loci remains to be tested but seems a not unrealistic scenario. Below, we give three brief examples of empirical systems that allow experimental manipulation and quantification of phenotypes relevant to the above hypotheses. While all of these systems have their limitations they represent a good starting point for empirical investigations of imprinting effects.

## Empirical approaches

### Mouse intercross

Several empirical approaches provide opportunities to investigate evolutionary mechanisms that contributed to maintenance of imprinting. Intercross populations can be used for genome-wide scans for loci showing imprinting patterns (Mantey et al., 2005; Cheverud et al., 2008; Wolf et al., 2008a; Hager et al., 2012a,b; Kärst et al., 2012). They are produced by breeding two parental mouse strains to obtain an F2 generation and then breeding this F2 generation to produce an F3 generation, which has a number of recombinations. If the F2 and F3 are genotyped and phenotyped, then quantitative trait loci (QTL) and imprinted QTL (iQTL) for a phenotype can be identified. By cross-fostering F3 offspring to foster mothers we can separate imprinting from maternal effects on offspring behavior and development, which may produce the same phenotypic patterns (Hager and Johnstone, 2005; Hager et al., 2008b, 2009; Leamy et al., 2008). Embryo transfer (Cowley et al., 1989) would reduce further pre-natal maternal effects but is in practice far from trivial to accomplish. Mouse model systems provide the opportunity to investigate phenotypes relevant to those assumed in hypotheses of imprinting such as example maternal provisioning (for testing the kinship and coadaptation hypotheses) or mating behavior (intralocus sexual conflict hypothesis). The advantage of using intercrosses is that effects of normal variation on phenotypes are examined rather than major genetic alterations. This contrasts with traditional methods of investigating imprinting, such as knockout (KO) or uniparental disomy studies which, although having provided a wealth of information (Schulz et al., 2008), lead to gross abnormalities that are unlikely to survive in a wild population and therefore unlikely to contribute to selection (Wolf et al., 2008b). With appropriately designed experiments that measure phenotypes relevant to imprinting hypotheses intercross analyses offer a promising approach to test specific predictions.

### Recombinant inbred strains and reciprocal heterozygotes

Recombinant inbred (RI) strains are produced by breeding two inbred strains, e.g., in mice or rats, for two generations. These recombinant litters are then inbred by brother/sister mating for >20 generations to produce a series of fully inbred lines, which are homozygous at every locus but have, across lines, a fixed pattern of two possible alleles (Peirce et al., 2004; Gini and Hager, 2012; Pollard, 2012).

**Figure 1:**
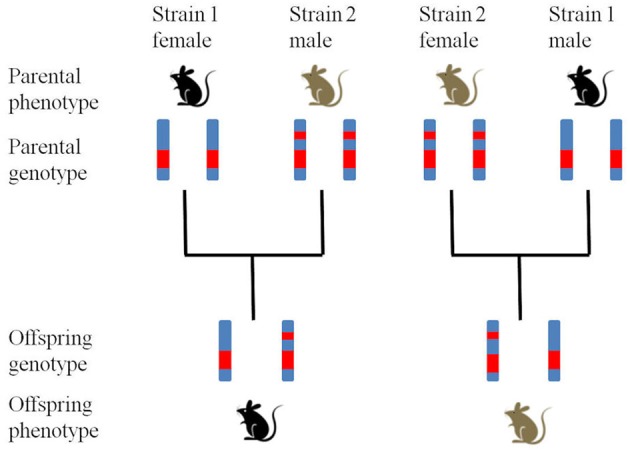
**Production of reciprocal heterozygotes.** Reciprocal heterozygotes are bred from two fully inbred parental strains (Strain 1 and Strain 2) to produce offspring with identical genotypes but different phenotypes (in this hypothetical example coat color showing a maternal expression pattern).

To explore parent-of-origin-specific effects potentially caused by imprinting, reciprocal heterozygotes (RHs) can be used (Figure 1; Ashbrook and Hager, unpublished). Specifically, the RH offspring born to heterozygous mothers should be genetically equivalent except for the mtDNA if, as is the case in RI sets, the original cross was done in only one direction (e.g., B6 female × D2 male). Therefore, differences in phenotype or gene expression will be due to parent-of-origin effects. NB. If mothers have different genotypes maternal genetic effects can cause phenotypic patterns that mimic those caused by genomic imprinting (Hager et al., 2008b). By backcrossing the RI lines to their parental strains, four ordered genotypes (two homozygotes and two RHs) can be produced for each locus, from which additive, dominance and imprinting effects can be determined. Again, using these animals in an appropriate experiment would allow testing predictions. The advantage of using RHs in RI lines is that the genotype can be reliably predicted which means that no further genotyping is needed and that experiments can be replicated by other groups collecting additional data that may jointly be analyzed. More generally, studying rodent model systems allows generating scenarios in which to test model predictions experimentally, something that is rather more difficult to achieve in human studies.

### Gene expression, transcriptomics and epigenome analyses

Several approaches can be used to determine allele-specific expression patterns. RNAseq allows the quantification of expression from each of the parental alleles and studies similar to Wang et al. (2011), in the placenta, and Gregg et al. (2010), in the brain, could be carried out in other organs such as the liver, kidney, or heart. Changes in these organs are likely to have little effect on the developmental or reproductive traits that are targeted in the kinship and coadaptation hypotheses and, as it has been shown that imprinting can be both spatially and temporally variable (Monk et al., 2006; Schulz et al., 2006; Fowden et al., 2011), these organs should have few imprinted genes, particularly in adults. For example, Cheverud et al. (2008) found evidence for much smaller imprinting effects on heart weight than, for example, on bodyweight. High levels of imprinting in these organs would pose a challenge to the established hypotheses of genomic imprinting.

Transcriptome measurements, such as RNAseq, in these rodent populations may provide a way of testing the hypotheses described, as a number of comparisons can be studied, for example imprinting in males and females (intralocus sexual conflict), the ratio of maternal to paternal imprinted genes (coadaptation hypothesis) and changes in imprinting over time and between tissues (kinship hypothesis). RNAseq methods can reveal novel imprinted genes by identifying loci with differential levels of gene expression between the two parental alleles.

More recently, new techniques have been developed to scan the genome for potential imprinting, for example by examining hyper or hypo methylation in the genome (Emes and Farrell, 2012; Xie et al., 2012). This has led to the ability to perform epigenome wide association studies (EWAS) in humans, where phenotypes, usually disease phenotypes, are linked to epigenetic marks. Studies so far have mostly been interested in methylation, profiling the methylome (such as Bell et al., 2010; Rakyan et al., 2011), however, newer techniques may allow the profiling of other epigenetic marks (e.g., histone modification) in the same samples (reviewed by Verma, 2012). Experimental challenges, in particular in humans, relate to cohort sizes, obtaining tissues at different developmental stages and difficulties in controlling for confounding environmental factors.

Clearly, different species are subject to different selective pressures, and therefore the evolutionary mechanisms found in rodents may vary from other species with different life histories, notably humans. In sum, testing alternative hypotheses for the evolution of imprinting is at present challenging as they are not mutually exclusive and each hypothesis may be applicable to explain imprinting at a specific set of loci. What is required are biologically relevant empirical tests of the scenarios outlined by theoretical frameworks. We believe that rodent model systems such as intercrosses and RI lines offer the best opportunity to test predictions in mammalian model systems.

### Conflict of interest statement

The authors declare that the research was conducted in the absence of any commercial or financial relationships that could be construed as a potential conflict of interest.
